# Castration of the Dog—Its Desirability as a Means of Checking the Sheep-Killing and Other Evil Propensities of the Species

**Published:** 1880-04

**Authors:** Alexander Hadden

**Affiliations:** N. Y.


### ART. IX.—CASTRATION OF THE DOG—ITS DESIRABILITY AS A MEANS OF CHECKING THE SHEEP-KILLING AND OTHER EVIL PROPENSITIES OF THE SPECIES.

BY ALEXANDER HADDEN, M. D., N. Y.

THE raids made by dogs upon sheep and other defenceless domestic animals, such as fowls, have been so frequent, and the consequent destruction of property throughout the United States alone has been so great, that almost every means that could be thought of to prevent them and still retain this useful animal as a part of the household has been devised and put into practice, but, as yet, with only partial success.

The chief preventives hitherto employed have been the following: The destruction from time to time of the animals themselves; the assessment of damages upon the owner of the culprit; the levying of a tax upon dogs, the revenue arising from which to be applied to the liquidation of all damages resulting from these raids; the breeding and employment of certain varieties of the species—namely, such as might be thought to be less prone to offend in the manner indicated, in preference to other varieties more likely to offend.

As the statistics show, none of these expedients, nor all of them together, have yet been found effectual in preventing the evil. The Commissioner of Agriculture, in his report for the year 1865, makes the following startling statement in regard to the ravages of dogs upon sheep in the United States; and this, too, comprises only the returns from 373 counties of 23 States. Number of sheep killed in that year, 77,854. From this he estimates the whole number of sheep killed by dogs in the United States during the same period at, in round numbers, no less than 500,000 head, and the loss to the growers at $2,000,000. Since

1865 no reports have been made, but there is good reason to believe that the ravages since then, and the consequent pecuniary loss, annually, have been no less.

The offence alluded to is about the only one of any consequence with which the dog, the companion and friend of man, is justly chargeable. And it is not upon sheep alone, but also upon calves, and even upon the fowls of the barnyard, that he makes his sallies. All this, of course, helps to swell the aggregate of the enormous loss entailed. To prevent this, if possible; to remove or subdue this objectionable trait in his character—a vice, and which, indeed, seems to be almost the only remaining vice pertaining to his wild state—and to more thoroughly domesticate the animal, should now be our aim. In order to accomplish an end so desirable the most fully and completely, we venture to suggest that no method that can be devised will do this more effectively than castration: castrating the males and spaying the females of all such as are allowed to run at large.

Upon inquiring into the matter, we find that dogs are not driven to undertake these raids by hunger or by any other such uncontrollable propensity or desire, but are actuated wholly by a spirit of mischief, which seems to be an incidental remnant of their wild state. Furthermore, we discover that the dog never goes upon one of these raids alone. He always seeks a companion of his own species, and sometimes the animals go in packs. It is noticeable that these raids are almost invariably preceded by the intimate association of neighboring dogs.

Castration produces a change—if we may be allowed the expression—in the moral character of the animal. The objects of his attachment are now different. Whereas, before, he wandered from home, seeking the companionship of others of his kind; now, he becomes more firmly attached to his home, and more dependent than ever upon his master.

I put this method—that of castration—into practice about six years ago, and have always suggested and advocated its employment. The animal then operated upon was a watch-dog, and only a mongrel pup. He did not grow fat and sluggish, as all authors writing upon the subject state that *castrati* will do, but he still continued to be bright and active as he was before, and remarkably docile. One of my friends, who owned a very fine shepherd's dog which he has had castrated, thinks that the operation has been in all respects a success, and states that the animal has retained all his useful qualities as a farm dog. Dr. C. Burden, veterinary surgeon, says that one of the best rat terriers that he ever knew was a *castratus*.

We have a letter from a gentleman living at Mount Pleasant, Wayne Co., Pa., Mr. Arthur Stevenson, who may justly be regarded as an expert in all that relates to the dog. He says in the letter referred to: "I have, during a period of many years, operated on a great number of dogs, and I have never to my knowledge killed a dog by so doing. And I can truly say, that I have never known a dog whose habits were not materially improved by the operation. I think they will last longer and do better service." On being interrogated, he states further, that he never has known a castrated dog to have hydrophobia, or to engage in killing sheep. Not because a castrated dog might not contract hydrophobia the same as another if bitten by a rabid animal, but he is not so apt to associate with other dogs, hence his chances for taking the disease are reduced to a minimum. Canine madness is thought by some authors to originate in ungratified sexual impulse; hence, as our correspondent states, the disease cannot originate with a dog that has been castrated. And although, as stated, he has never known a castrated dog to kill sheep, he is yet willing to allow that such might do so, if the habit had been formed previously. He therefore recommends that all dogs intended for domestic use should be castrated at an early period of life; say when not more than six months old, before they have had opportunity to contract any vicious habits.

Mr. Darwin, in his Researches into Natural History, tells us that in the *Banda Oriental*, in South America, it is a common thing to see flocks of sheep guarded by one or two dogs at a distance of some miles from any house. After describing the method of training the dogs to perform this service, he goes on to say: "The puppy is, moreover, generally castrated, so that when grown up, it can scarcely have any feeling in common with the rest of its kind. From this education it has no wish to leave the flock, and just as another dog will defend his master, man, so will this the sheep." We do not quote our author at length, when he describes minutely in what the education of these dogs consists, but content ourselves with stating the fact of their castration, which we regard as the main fact in this narration, and the one thing which, to our mind, makes the other steps in the process effective.

The statement made above, that the raids upon sheep and other defenceless animals are made only by the male canine, is corroborated by the testimony of Mr. Wm. A. Conkling, Superintendent of the Zoological Department of Central Park, New York. When these marauders frequent in greater numbers than usual the enclosures where deer and certain other animals are kept, an attempt to abate the nuisance is sometimes made by poisoning the offenders. Among the dead bodies found afterwards lying about, Mr. Conkling states that he has never yet found a bitch.

To the reasons already urged for the castration of the dog we might add further, that in the emasculated condition he makes a less objectionable house pet, and is in this respect far superior to the bitch. He will also live to a greater age. As a watch-dog he is not likely to be decoyed away from the post of duty by a bitch in heat, or by any other dog. This is a well-known stratagem which thieves and burglars frequently employ. Obscene street scenes would be avoided. There is a law to prevent stallions and rams from going at large. In the interests of decency the same law should, we think, be extended also to the dog, if the other sufficient reasons which have above been urged do not prevail.

